# Remarks on the Pathology and Treatment of Intermittent Fever

**Published:** 1833-03

**Authors:** John Bell


THE LONDON

### Medical and Physical Journal.

409, VOL. LXIX.] MARCH 1833. [81, *New Series.*

"For many fortunate discoveries in medicine, and for the detection of numerous errors, the world is indebted to the rapid circulation of Monthly Journals; and there never existed any work to which the Faculty in Europe and America, were under deeper obligations than to the 'Medical and Physical Journal of London,' now forming a long but an invaluable series."—RUSH.

### ORIGINAL PAPERS.

### INTERMITTENT FEVER.

### *Remarks on the Pathology and Treatment of Intermittent Fever.* By JOHN BELL, M.D.

THE influence of hypothesis in the treatment of intermittent fever, has been of long duration and unequivocal injury. Reformation can only be brought about by our consenting to remove this disease from the list of exceptions, and to subject it to the same rules of analysis which are deemed valid in other maladies. For this purpose, we must cease to regard it as originating from a specific cause, and cured by a specific remedy. The product of atmospherical vicissitudes and distemperatures of seasons, intermittent fever, will be found akin to all the varieties of gastro-intestinal inflammation, as well as to hepatitis, splenitis, rheumatism, catarrh, chronic pleurisy, pneumonia and bronchitis, peritonitis and its attendant ascites; by some one or many of which it is often sustained. Its type is that often assumed by all the phlegmasiæ and hemorrhagies, and the innumerable tribe of maladies called nervous, having their origin in a lesion of some part of the cerebro-spinal axis. There is scarcely a disease in the nosological catalogue which has not at times preserved a distinctly periodical character; its invasion being marked by a chill, and the subsequent fever terminating in sweat. I have seen fatal inflammation of the liver and gangrene of the lungs exhibit four distinct tertian paroxysms; and the instances of periodical fever originating in and kept up by irritation of the stomach, and even bladder, are of common occurrence and familiar observation. It is not necessary for me to repeat the opinions which I advanced on former occasions respecting the erroneous views of the origin of periodical fevers,* and of the unfortunate therapeutical treatment based on them. My purpose will be answered by thus briefly reminding the reader of the periodicity of inflammatory, hemorrhagic, and nervous diseases; and that, consequently, no single remedy nor uniform course of treatment ought to be recommended for the mere type, without reference to the nature of the functional disturbance and the extent of organic lesion. Clear and obvious as this principle may seem to us, the practice has been too generally at direct variance with it. An individual must often be content to suffer from oppressive headach, or pain and heat of the stomach, or of one or both hypochondriac regions, or dysenteric gripings, thirst, dry pain, and general uneasiness; if with these states be associated periodical paroxysms, called intermittent fever. Supposing the stomach and bowels to be well emptied and cleansed, bark, in its several forms, is poured in, with the accompanying assurance, on the part of the attending physician, that this will cure all his complaints, by preventing the return of the chill. Not unfrequently, however, the promise proves to be fallacious: the symptoms are unmitigated in violence, and the chills are then declared to be very difficult to remove. What is the reasoning of the physician in this emergency? He meditates on all the tonic remedies which are alleged to have the power of curing intermittent fever: he selects arsenic from the list, and administers it to his patient. Persevered in for some time, this poison breaks up the periodical character of the disease; there is no longer a recurrence of chills, and the invalid is congratulated on his escape from disease. But health is slow in its approaches; he digests badly, has pain in his stomach, with occasional nausea and disturbed bowels; or, in a more favorable state, he complains of dull, fixed headach, pain in the side and right shoulder, thirst, and scanty urine. In place of these there will be occasionally a tumid abdomen, œdematous extremities, cold skin, and sluggish pulse. Reference being had to the want of appetite and bad digestion, some of the milder tonics are administered; or, the disorders persisting, visceral obstruction is suspected, and an alterative course of mercury is prescribed. The first palliate, perchance, some uneasy sensations for awhile; but fail to prevent, if they do not aggravate, the confirmed dyspepsia now entailed on the unhappy invalid. The second, after being carried to the extent of producing salivation, seems to

* See the papers on Periodicity and Miasm, in Dr. Chapman's Medical and Physical Journal, and in the North American Med. and Surg. Journal.

remove or suspend the enlargement of the liver or spleen, and carry off the effused serum in the cellular tissue, but in a few days after, especially if he be exposed to an easterly wind or night-air, the chill returns, and the patient is doomed to the same round of tonics and alteratives as before, with a still stronger conviction in the mind of his physician that the long duration of the disease renders it more than ever indispensably necessary to husband his strength by nourishing diet, porter, wine, &c. Winter now approaches, and a troublesome cough, with pleuritic stitches, is superadded to gastric or hepatic derangement, or both.

In drawing this picture of the progress of intermittent fever, its complications, effects, and treatment, I am not conscious of exaggeration or misrepresentation. The dominant idea in the mind of the physician, for one in his case will represent a large class, is debility, and the necessity of tonics exclusively for its cure. How powerful the force of associations! The same state of the circulation and digestive passages; the pain in the head, side, or chest, which, in other circumstances, would be met by the remedies for inflammation, are here, in consequence of their connexion with periodical fever, regarded as mere accidents, and adventitious and secondary symptoms, which we must not attempt to remove by sanguineous depletion, for fear of enfeebling the patient: but this fear would be just as well founded in periodical hemorrhages, epilepsy, gout, and rheumatism.

In intermittent fever, as in all the diseases of autumn, the stomach is much disturbed in its functions, especially just before and during the paroxysm. Nausea, vomiting, thirst, and heat of the epigastrium, are common symptoms on the subsidence of the cold and coming on of the hot stage, and are often rendered more violent by the stimulating draughts taken during the period of the chill. Here begins the deviation from the principles which govern us on the attack of other diseases. In this latter case we are wary in prescribing any stimulant or irritant beyond external warmth or a simple warm diluent, however great may be the rigor, well knowing that the subsequent reaction will only receive additional aliment from our efforts to rouse the patient from the state of depression in which he is thrown during the first invasion of the disease. Whereas, in the chill of an intermittent fever, the popular practice, too readily countenanced by the profession, is to swallow all kinds of heating drinks, alcoholic, cordial, and vinous. The hot stage having now come on, aggravated by these imprudences, we are counselled to use various mixtures, with the intention of quieting the irritation of the stomach, and bringing on diaphoresis. Laudanum, the alkalies, and carminatives, constitute the basis of most of these prescriptions: all of them are more or less stimulating, and in no one of them can we repose confidence in gastric irritation, or gastritis; for such, in fact, pending the paroxysm, is the true state of the stomach. Experience, corroborating the inductions of theory, daily teaches us that the fewer drugs given, and the simpler, cooler, and blander, the drinks, the more shall we have it in our power to control vomiting and pain, heat and distress of the stomach, and associated disturbance of the head. These failing, bleeding from the arm, cold affusion, and, in more obstinate cases, leeching and cupping, are found to be the most efficient means of quieting an irritable stomach. The reaction in the hot stage consists not only of increased gastric sensibility, but of accelerated circulation and excessive capillary and nervous excitement. The pulse is frequent, full, and vibrating, and all the symptoms evince a general disturbance of the organs of assimilation and secretion, including the stomach, liver, kidneys, lungs, and skin. Of this the vomiting at the beginning, and the sweat and urinary deposit at the termination of the hot fit, afford abundant evidence.

The call for moderating excessive excitement is not less imperative here than in other maladies, distinguished by similar symptoms, however variously named. We are well aware of the general principle on these occasions, that the risk of subsequent languor and engorgement, or even disorganization of the organs and disturbance of functions, is greater if the excitement be allowed to wear itself out by our abstaining from those means calculated to moderate it.

In the case before us, we have, superadded to gastric irritation of the stomach, a morbidly-exalted action of the other great viscera. Of course, in addition to the indications furnished by the former, we have those supplied by the latter, to direct us to the use of the lancet and the application of cold. In fact, the treatment here is identical with that in every febrile paroxysm of any intensity, whether it be of regular, or spurious, or malignant, intermittent, or remittent, yellow, or bilious. It is true that a temporary crisis will generally take place in regular intermittents, without any interference on the part of the physician; but every paroxysm, especially if neglected or mismanaged, predisposes the subject of it to complications and aggravations, at each successive return; and the stomach, liver, and head, from being periodically affected, are after awhile permanent sufferers.

Bleeding in the hot stage of intermittent fever, thus pointed out by inductive reasoning, has been proved by experience to be on many occasions decidedly beneficial. It is now many years since, whilst yet a student in Virginia, it became my duty to see and occasionally prescribe for a young man of a thin spare habit of body, who had been much reduced by repeated attacks of intermittent fever. Bark and arsenic had been administered in vain. Influenced by the recommendation of Senac, whose work on Intermitting and Remitting Fevers I had just perused, I opened a vein in the arm of my patient during the next hot fit, and took away a pint of blood. The relief was immediate; the force of the paroxysm soon subsided; the apyrexia was complete; and a few doses of bark were sufficient to prevent the next fit. He speedily recovered his health and strength, and remained clear of intermittent fever. From that time to the present I have not hesitated to use the lancet in every case of periodical fever, in which either the apyrexia was not so complete as to leave the patient entirely clear of all gastric and cerebral distress, or in which the paroxysms had been of frequent recurrence, and untractable under the use of bark. I have usually preferred, when the choice was in my power, bleeding during the hot stage to doing it in the apyrexia; but the experience of every additional season convinces me that, in this latter state also, the employment of the lancet will realize all our best hopes.

Both as adjuvant to and substitute for the lancet in the hot stage, cold affusion or immersion is entitled to special notice and favor. The tranquillizing and pleasurable effects of cold water to a patient, in the burning heat of fever, must be actually experienced by an individual before he can properly appreciate them. The chief obstacle to the use of this remedy, now that the prejudices of ignorance are in a great measure dissipated, consists in the want of suitable conveniences for bathing, among the larger portion of those who suffer from intermittent fever. Seldom, however, shall we be unable to procure a large tub for the patient to stand or crouch in, and one or two buckets, the contents of which are to be poured on him. Sponging the surface of the body may be had recourse to for the purpose of allaying the morbid heat in the hot stage; but it is an indifferent substitute for immersion or affusion, unless in particular cases and circumstances of age, sex, and states of mind, which we can readily conceive.

Coinciding with the effects of cold water, externally applied, will be those from its internal use. In lieu of water in its simple state, we may give it slightly acidulated with lemonjuice, or cream of tartar; or, if the stomach be much distressed, gum arabic will be a useful addition.

Having by these means brought our patient through the cold and hot stages, the sweating will seldom be excessive or enfeebling, and the succeeding period will more probably be, not in name, but in fact, that of apyrexia. Should it prove such, that is, should the tongue be moist and but little loaded, and the skin soft, we can then, with every prospect of entire success, give the bark or its salts in full doses at short intervals, until, in the revolution of time, the epoch arrives at which the next paroxysm would probably have come on.

It will be seen that I am not so exclusive as to recommend bloodletting and cold as remedies, which, if used during the hot stage, are of themselves capable of curing intermittent fever. But thus much I can aver, that the febrile exacerbation will be briefer in duration and diminished in violence, the succeeding interval freer from all unpleasant sensations, and the return of the paroxysm postponed, and, when it does come, rendered less violent, after the use of the lancet.

If the period following the paroxysm be not one of complete apyrexia, and there remains some heat and tenderness in the epigastric region, with a furred tongue and red at the borders, we shall, for the most part, only increase the general discomfort of the patient by administering bark and other tonics, wine and rich food; even though, by this treatment, we prevent the coming on of the next paroxysm. The case is to be treated in all points as we would treat gastric irritation under other circumstances, viz. by simple drinks and very light bland nutriment, and moderate exercise, with pediluvium or the warm bath.

I do not speak of the state of the pulse during the apyrexia, because I consider the indications which it furnishes as very fallacious. It is most common to find it exhibiting under the finger a certain degree of activity, compounded of fulness with vibration, in the progress of intermittent fever: but this resistance furnishes hardly any reason for depletion, if the stomach be sound, tongue moist, and skin soft; nor would its smallness and feebleness be an argument for prescribing the bark, or abstaining from general or local depletion, or both, if the stomach were much distressed, the head pained, the hypochondria tender and tumid.

I hold it as a fair inference from pathological phenomena, justified by large experience, that, in this last-mentioned condition of things, bloodletting, in the interval of intermittent fever, is as safe and efficacious as in any other form of disease whatever. It removes phlogosis of the mucous membranes of the digestive and pulmonary apparatus, so often combined in the subjects of intermittent fever, at the beginning of winter and spring; as well as prevents engorgement of the liver and spleen, and aids in removing it if once formed. The prevention of the paroxysm becomes, after the use of the lancet, a most simple affair. Pounds of bark or drachms of quinine have been given in vain to cure an intermittent fever, which has in a short time completely yielded to a few doses of these medicines, when their administration was preceded by a single bleeding. Of this fact I have had numerous examples: and so much were the patients themselves surprised at the prompt relief afforded, that, overlooking the bleeding, they imagined the quinine pills which they had taken afterwards, to be a new medicine, possessed of uncommon and peculiar powers. What is said of general bleeding will apply in many cases to topical depletion, by means of leeching or cupping. The predominance of gastric symptoms would point out the propriety of applying leeches over the epigastrium; the complication of pulmonary ones would indicate the use of cups to the chest. Whether bleeding from the arm shall precede these operations must depend on the intensity of the visceral disease and the circumstances of the constitution, and general habit of body of the patient. The former will of itself most generally meet our expectations, if a regulated and reduced diet be persevered in, so that the stomach, ceasing to be offended, may recover from its state of irritation.

By an adoption of this plan, if we do not entirely remove the periodical paroxysms, we at least reduce them to their simplest element, or that of nervous irritation. Nutrition even may now go on, and the individual's health not be materially deteriorated. But, as before remarked, we need not subject him to the experiment. Bark, or some kindred tonic, will now readily suffice to accomplish a perfect cure. Very different is the result, when, without regard to the capillary system and the membranes which it mainly composes, we are satisfied to destroy the periodicity, and nurse ourselves with the illusion that we have removed the disease, because we have suspended the return of pure nervous irritation.

Of other remedies during the interval of intermittent fever, I deem it needless to say much, because every year diminishes for me the number, by my discovering the greater success attending a few than many. Were I required to speak of any one medicine laying claim to pharmaceutical activity, which I have used with most advantage in a period often necessarily intervening between the use of the lancet and the employment of the salts of quinine or the bark, I should unhesitatingly instance the blue pill. Not unfrequently, when the disease prevailed so extensively in the city and its vicinity a few years ago, I gave a five-grain pill of the blue mass at night and the cinchonic preparations during the following morning, with the effect of promptly arresting quotidian fevers, which had not yielded to the bark alone. The practice is equally applicable to tertian fever. I must add, however, that when this course was followed, I had not recourse to the lancet with the same freedom as subsequently. Bloodletting simplifies the practice so much, that we are enabled in the majority of cases to dispense with any intermediate medication between it and the use of the bark. I have indeed prevented a return of the paroxysm by giving five-grain doses of the blue mass three times a day, after bleeding; but, although this will in some instances be entirely successful, I am far from recommending it as a general rule of practice.

With still greater hesitation would I encourage a perseverance in the use of the mercury until salivation had been produced. I have seen the fits suspended by the mercurial disease, but I have never known a patient to escape from a speedy relapse, if the cinchonic preparations had been withheld. Twice in my own case, when suffering from the disease in China, in the year 1818, I arrested the disease by slightly touching the gums; but a relapse soon followed, on the very first exposure to the night air in one case, and to an easterly wind in the other. Removal to the seashore at Macao, and a few doses of bark, very promptly restored me to comparative health, and allowed me, at the proper season, to discharge my duty as the medical attendant on the American shipping then lying below Canton.

In illustration of the modifying influence of climate on the progress of disease and the effect of remedies, it is but right for me to state that, during the months of September and October, when there were most sailors in that port, and when the intermittent fever was rife, I was not baffled in a single intance in my attempts to cure, by a very simple treatment, those subjected to this disease. Sometimes evacuations were premised; at other times they appeared unnecessary. The chief reliance was on the bark, in drachm doses, administered at intervals of every two hours, beginning at the expiration of one paroxysm, and continuing the medicine until about an hour before the epoch at which the next would have come on, if no curative measures had been adopted. Rarely did a patient suffer from the third paroxysm of a quotidian, or the second of a tertian; nor was he long in being restored to ship duty.

It is not easy for me to pronounce to what extent these men would have suffered from relapses had they remained in Canton during the winter, or what was the state of their health in the following spring at home; but I have reason to believe that relapses with them were not unfrequent. Be this as it may, I have not found the above simple practice answer in our own country, in which, from the excesses and vicissitudes of climate, visceral inflammation and engorgement are so common; while the same relief is not afforded as in tropical regions by copious cutaneous transpiration.

Occasionally we hear of practitioners relying exclusively on the use of the cold bath for the cure of intermittents. A knowledge of the directly sedative effects of cold, and observation of its power of reducing febrile action to the normal healthy standard, as in the hot stage of intermittent fever, will make us slow to direct it during the interval, when there is often little or no superfluous excitement, and the predisposition to chill is manifestly great. It is hardly wise to irritate a paroxysm of fever by subjecting an individual, whose nervous system is rather enfeebled than excited, to a cold bath. He is necessarily chilled; has some rigors; and in the most favorable state a subsequent glow and reaction. Nor does the disturbance end here: pains in the limbs and head, and languor, are often complained of by those who use the cold bath, when the system is not above the natural level of excitement. From these premises, not a little strengthened by experience, I should feel inclined to regard habitual cold bathing in the interval as a hazardous remedy, and rendered often mischievous by the prevalent errors regarding its modus operandi. Very different are its effects when used in the hot stage of intermittent, or in the more permanent capillary excitement of gastro-cerebral, fever, usually denominated typhus. The morbid excitement of the sanguineo-nervous structures, entering into the composition of the membranes, and chiefly instrumental in the secretions, including that of caloric, is abated and often entirely removed; the patient is rendered tranquil, and enjoys a pleasant slumber unbroken by the former irritations of heat and thirst. Just in proportion as the state of the patient during the interval approaches to that exhibited in the hot stage will cold bathing be useful, but not otherwise. Hence, if there be a steady dry heat of the skin, frequent pulse, with thirst, and little or no appetite, we shall derive good effects from cold affusion in the period between the paroxysms. This remedy is not therefore, as often taught, akin to bark; the two stand opposed to each other in their effects, and their use is only properly called for under different and opposite circumstances: the one to allay morbid irritation and inflammation; the other to exalt and strengthen parts already feeble.

A review of the effects of arsenic in intermittent fever, that is, on those labouring under the disease who have taken it, would, on calm and dispassionate reflection, induce us to wish that its use had never been proposed. The amount of mischief which it has produced must have been excessively great, and exceeding the good which it has been alleged to do, in the same proportion in which cases, where it has been given in ignorance of its operation and the state of the system, have exceeded those where it was administered with all the reservations and restrictions which could be suggested by cautious observation. We are told that it has cured when the bark has failed: but I think it has been sufficiently shown that if the bark fails we have other duties to perform towards our patient than hunting out fresh tonics. When thus unsuccessful, we shall find the stomach irritated and perhaps inclined to phlogosis, or there is a chronic hepatitis and a tendency to dropsical effusions. Now, most assuredly, arsenic, even if it arrest the chill, is not the appropriate remedy in these circumstances. If we persist in giving it, we do so at the peril of our patients, to whom we stand fearfully responsible for the chronic gastritis thus entailed on them. Three of the most obstinate cases of disease which were presented to my notice during last year, were of persons who had used Fowler's solution for the cure of intermittent fever. One was a young man from the country, who had been cured of the chills by this medicine, but who suffered greatly from pain and heat of the stomach, which had supervened since be began using the solution. Two bleedings and reduced diet made him more comfortable; but I was not permitted to see him entirely recovered, in consequence of his return to the country. The next was a young female, who, when I saw her, was greatly distressed by irritability of the stomach, with fixed pain and frequent vomiting; her pulse was hard and active; the appearance of the complexion and other symptoms induced a belief that she laboured also under hepatic disease. Upwards of two months elapsed before her digestive powers could allow of her using any food at all stimulating. During that period she was frequently bled from the arm and leeched over the epigastrium. The blue pill was administered, and after awhile the sulphate of quinine; but, as I soon found, however, the morbid state of her stomach aggravated by their use, I was content, at last, to rely on depletion, general and local, as above, and occasionally a mild laxative and diluents. Under this treatment she so far recovered as to justify the use once more of the quinine; by which, finally, the disease lost its periodical character, and strength and health followed. The third case, also of a female, was characterized by nearly the same symptoms, but they were of less duration, and were finally removed by several bleedings, and by the use of the quinine.

It would be well for us to bear in mind that there are two modes of poisoning. The one sudden and acute, resorted to in moments of temporary insanity or impious despair, for the purposes of speedy self-destruction, or with malice prepense to take away the life of a neighbour; the other is a slower process, practised by empirics, when they persuade the ignorant and credulous to swallow their nostrums, and, shall we add, by regular physicians when they direct, without due deliberation, their patients to use arsenic, corrosive sublimate, and some other half dozen of heroical medicines.

As it is not my intention to write a systematic essay on intermittent fever, I forbear to pass in review the long list of vegetable and mineral substances which have at different times acquired distinction for the cure of the disease. Of their remedial power I am fortunately ignorant, for the reason that I have always found the cinchonic preparations, and of these the simplest kind, answer all my wishes, when had recourse to with the restrictions already pointed out. The facility with which the salts of quinine can be procured obviates all the difficulties which formerly impeded us in the free administration of the bark in substance. Inability to swallow and retain the former can no longer, as in the instance of the latter, furnish us with a pretext for seeking after new remedies.

There will no doubt be found many, to laud, as heretofore, the combination of bark with opium, serpentaria, the alkalies, &c. or with other bitters and astringents. With such I do not mean to cavil; but would simply express my conviction that if the stomach and system generally be not in a suitable state for the reception of the bark, we but deceive ourselves and injure our patients, if we think that any addition to it of kindred preparations, drawn from the class of stimuli and tonics, or even of antacids or acids, will render it safer and more efficacious. So, likewise, if the stomach be in a proper state for the ingestion of any tonic, we are safe in presuming it is so for bark and its salts. And again, though we may admit that a plurality of remedies, usually called tonics, are each of them capable of curing intermittent fever, we ought by fair induction to allow of the possibility of one condition only of the stomach, in which any of them can be advantageously used.

Having assured myself of the propriety of giving the cinchonic preparations, I begin their use immediately after the termination of the paroxysm; at which time the solution of the fever is most complete, the capillary excitement most subdued, and the stomach most in want of moderate nutritive and medicinal stimuli. Repeated at brief intervals, in full doses, the medicine will be more likely to ward off the expected ensuing paroxysm than if we were to delay its administration until a short time before the epoch of the chill, when, the sensibilities of the stomach becoming less, whatever is then taken in lies comparatively inert and unchanged, until the reaction in the hot fit. If, notwithstanding the favorable auspices under which the quinine was administered, the disease still preserves its paroxysmal character, and the stomach and head are distressed, I do not hesitate to suspend the use of the medicine, restrict still more the regimen of the patient, and, irritation continuing, to bleed from the arm or topically. After a timely pause of this kind, the quinine may be again directed with a success incomparably greater, on the score of expedition, comfort, and freedom from subsequent ailments, than would have followed a substitution of other tonics after its first failure.

By those who regard intermittent fever as purely a succession of febrile paroxysms at stated intervals, which return by the force of the habit, various internal and external stimuli have been eulogized for their property of warding off the chill, if taken or applied a short time before the expected return of this latter. Opium and blisters have been recommended in a more especial manner as fitted to answer the view. That they will singly and conjointly often fail we are well aware: but even when they succeed in producing the effects anticipated, we are far from having the condition of the patient materially benefited. In preventing a chill they do not on that account remove the internal visceral disturbance by which the system was being gradually brought to this point. Even as revulsives, their operation will be of too equivocal if not perturbating a character to ensure our confidence. It may excite surprise to see the epithet perturbating applied to opium; but those who have witnessed its first effects on the brain and nervous system, and secondary ones on the stomach, will readily understand how this medicine becomes so when untimely administered.

The principle which to my mind seems most desirable to be established in the treatment of intermittent fever is, that in each stage we use the remedies indicated by the group of symptoms at the time, without anticipating future possible evils from present relief on the one hand, or distant good by aggravating actual distress on the other. By a neglect of this important principle, we often make the physician a timid observer, in place of an efficient actor, when he refuses to relieve the sufferer from cerebral, pulmonary, and hepatic congestion, and disorganizing membranous inflammation, by copious depletion, for fear of a little understood state of debility being afterwards induced. Changing, at another time, his character, he will be seen urging the use of irritating stimuli on a person already tormented by burning heat and thirst, and general capillary excitement in the second stage of remittent gastric fevers, for the purposes, as he alleges, of giving him strength, and enabling him to digest and be nourished.

The advocates of bloodletting in the cold stage of intermittent fevers admit the correctness of the above principle. They contend that the operation is both justified and called for by the condition of the viscera at the time. The question must be determined by similar arguments and facts to those which have been adduced in favour of, or opposition to, direct depletion, in the stage of oppression and chill in other maladies. The physician is very rarely called upon to give advice before this stage or period of invasion is passed, and reaction has taken place. He is generally well satisfied indeed if he be allowed to act before this latter has been of long duration, and in being thus favoured, he considers himself as attacking the disease at an early period. Deducing our opinion from what is presented to us in the phenomena of the first stage of concussion of the brain, we should say, that, at the beginning of a febrile attack, marked as it is by chill, rigors, listlessness, languor, disinclination to move and think, the nervous system is alone affected, and in virtue of its debility, the secretions, as well of caloric as of different fluids, are suspended. The sanguiferous system is as yet a stranger to the disease, and only becomes interested in proportion as the returning excitement of the nervous system communicates to it fresh stimulus. If prior to this stimulation being transmitted from the nerves to the heart and capillaries, that is, before the disappearance of the chill and cold stage, we abstract blood, it can only be with the intention of diminishing the amount of this fluid, and thereby easing the vessels when the hot stage, or that of reaction is arrived. Accordingly, Drs. Mackintosh and Ridgway, apparently ignorant of Dr. Rush's recommendation many years ago, to the same effect, tell us, that by bleeding in the cold stage of intermittent fever, the hot and sweating stages have been prevented.

In two cases in which I adopted this practice, the result was not of such a favorable nature. One was evidently benefited; but neither in this nor the other was I dispensed from the necessity of subsequent bleeding before the disease was arrested.

To these I ought to add a third, which, from its rare occurrence and the formidable nature of the symptoms, merits a more particular notice. It was of a young mulatto man, who had been confined to his bed for three weeks by gastric remittent fever. The paroxysms came on at irregular intervals, and were always marked by a frequent and rather full pulse, acrid heat of the skin, especially over the abdomen, and a burning thirst. Frequent bleedings from the arm and cupping over the abdomen had been practised; purgatives of a saline and mercurial character were occasionally administered, which gave some relief at the moment, but always left the stomach and abdomen more tender to pressure, and the skin hotter to the touch. During nearly the whole time the tongue was loaded in the middle with a whitish yellow coat, while its borders and tip were red and shining. After the expiration of the above time, convalescence seemed about to be established; the pulse was nearer a natural standard, thirst less urgent, and temperature of the skin, except over the epigastrium, of an ordinary nature. Pressure on the abdomen rendered the pulsations of the aorta very perceptible. The patient gained very little strength, although he was allowed light animal broth and farinaceous food. Visited in the afternoon of September 17th of last year, I found him in a state of great apathy, with an inclination to dose. The pulse was not materially altered, nor was there any other new symptom. A blister was directed to the back of the neck, and a laxative of rhubarb and magnesia at bedtime. At eleven o'clock p.m. I was sent for in great haste, and on my arrival found the patient in a state of complete coma, utterly insensible to all objects of sight, sound, and touch; his limbs, at first extended, remained in whatever position they were placed; the pulse was barely perceptible, and the breathing very slow. It was impossible to make him swallow any thing, or to elicit from him the slightest evidence of consciousness. On applying my hand to the epigastrium, I could feel the abdominal aorta beat with considerable force; so also did the carotids. The contractions of the heart were frequent, and laborious. The blister had been put on, but no medicine taken. Sixty leeches were now applied over the epigastrium, and sinapisms to the extremities. After the leeches had begun to fill, the pulse lost somewhat of its extreme tenuity, and by the time they were detached, it had regained its natural volume, was soft and easily compressible. The patient at this time began to move his eyes and the muscles of his mouth and face; he turned a little towards one side, yawned, and stretched himself. The extremities were still cold and unaffected by the sinapisms. Before all the leeches were removed, the skin became moist in places; and finally a sweat covered the face, trunk, and limbs, with the exception of the hands and feet. Enemata of tepid water were administered at different times through the night. In the morning, though languid, he was partially sitting up in bed, by leaning on his elbow, helping himself to some light nutriment. In the afternoon of this day he experienced some rigors, which disappeared in the evening in moisture on the skin.

On the evening of the following day, 19th, by eight o'clock, he was in nearly the same state as on the 17th, being completely comatose. Cups in large numbers were now applied to the temples, and over the abdomen, so as to detract about ten ounces of blood. The effect was most salutary, and the recovery even more prompt than from the first attack. Enemata of cold water were given on the present occasion.

An examination of the symptoms of the case on the morning of the 20th, as presented by the pulse and skin, seemed to justify the use of the quinine, from which the furred and chapped tongue on the preceding days had deterred me. A minute inspection of this organ now showed me that under this dry and cracked coat it was pale, and rather thicker than natural. This appearance was readily recognizable by looking at the tip and sides of the tongue. A solution of the sulphate of quinine in water, ten grains to the ounce of fluid, was directed. Of this a tea-spoonful was taken every hour until the afternoon. There was then a very slight exacerbation. The medicine was resumed on the following day, and continued for several days. The patient was thenceforward clear of paroxysmal attacks, and gradually and regularly regained his strength and health.

Here was an extreme case, in which the coma, evidently a substitute for the cold stage of intermittent fever, was relieved on both occasions by a free abstraction of blood. The subsequent reaction and distress were very inconsiderable, and did not on either occasion prevent the patient from sleeping quietly during the remainder of the night.

Some of my readers will doubtless be surprised at my silence on the subject of emetics and purges in intermittent fever. They may feel inclined to regret this omission the more, as to them it will seem an evidence of the predominance of French doctrines. Before replying to this supposition, I would recommend to those who are so backward in giving credence to the weight of contemporary authority, a short essay in a small book by CLEGHORN. The chapter on tertian fevers, in his "Observations on the Epidemical Diseases of Minorca, from the year 1744 to 1749," to which I allude, is the production of a patient and experienced observer. Of nearly a hundred persons who perished in malignant tertians, and who were examined by him after death, the contents of the abdomen were in all greatly affected with congestion and inflammation. It was natural then for him, when called in the beginning of tertian fevers, to take away some blood from people of all ages, unless there was a strong contra-indication; and farther, if a violent headach, and obstinate delirium, and great heat or pains of the bowels, were urgent, within a day or two he repeated the bleeding, "by which seasonable evacuation the vehemency of the paroxysms is somewhat diminished; the apyrexias become more complete; the operation of emetics and cathartics is rendered safer and more successful; and the terrible symptoms which often make their appearance about the height of the distemper, such as raving, sopor, difficulty of breathing, inflammations of the abdominal viscera, &c. are either prevented or mitigated." The author adds some cautions in the use of the lancet, if the disease had continued some time, and the patient was very feeble; or if the paroxysms of the disease were attended with profuse evacuations, whether by vomiting, purging, sweating, or a hemorrhage from the nose. In all such cases, however, I have bled with advantage, after I had convinced myself that there was protracted irritation or engorgement of any of the viscera. CLEGHORN tells us that he commonly opened a vein at the beginning of the hot fit; but, if this time had passed before his being called, he bled in the evening when it abated or went off, that he might be at liberty next day to make use of the remission or intermission, which commonly happens in the morning, to evacuate the first passages.

As explanatory of my own views in withholding the use of emetics in intermittent fevers, and, at the same time, as a reply to the cavillers against what they are pleased to call the refinements of French practice, I will venture to extract, verbatim, the entire paragraph in which their own classical Cleghorn, one of those whom Rush is said to have called medical apostles, gives his opinions on the subject.

"It is a controverted point whether it is best to discharge those noxious humours by vomit or stool. At first view vomits seem to be most eligible, as they quickly empty the superior part of the alimentary tube, which appears to be the principal seat of the morbific matter. But it must be considered that whatever irritates much, and produces violent commotions, ought to be avoided in the present case. Cave ne inducas effervescentiam biliosorum is a caution given by Avicenna; and the Spaniards, as well as the Italians, if their physicians may be credited, cannot well bear rough medicines of any kind. (Vid. Bagliv. lib. i. c. xv. § 5.) Besides, the inflammations of the bowels, too frequently accompanying tertians, are exasperated beyond expression by the strong contraction of the diaphragm and abdominal muscles in the operation: and if the spleen or liver is disposed to become putrid, which is no uncommon case in these fevers, it is needless to point out the dangerous consequences that may result from the repeated efforts of vomiting. For which reasons, mild purgatives, though less powerful remedies, are the safest, and therefore to be preferred in the generality of cases. Those which I have found most beneficial are senna, manna, cremor tartari, but above all the sal catharticum amarum, (Epsom salts), which neither gripes nor disturbs the body, and seldom fails of having the desired effect in a few hours, a circumstance of great moment where the intervals are short. But, if vomits are to be used, they should be given in the beginning of the disease, before repeated paroxysms have brought on inflammations, or too much dissolved the texture of the blood, taking care that the operation does not interfere with the fit, lest some sudden mischief should arise from the united shocks of the remedy and the disease."

If we except a few words, indicating a belief in the humoral pathology, the preceding advice and cautions are just such as might be expected from an enlightened pathologist of the present day. The only justifiable season for giving an emetic in intermittent fevers, must be at the very commencement, and in those cases only in which we have every reason to believe that indigestible substances, as of fruit and vegetable matters, have been taken and still remain in the stomach, a source of irritation and distress.

In reference to repeated evacuations, Cleghorn, after telling us that he did not carry them as far as formerly, adds, that, as he seldom omitted to bleed and purge once or twice, he rarely repeated either operation oftener. He instances worms in the first passages, which sometimes accompany tertian fever, as another inducement for purging in the beginning. In other words, we are to empty the intestinal canal of the residue of digestion and other obviously foreign matters, which had not escaped, or had been long retained, before the coming on of the disease. Then, if it be clear of inflammation, we can give the bark immediately, with great advantage. If phlogosis be present in any part, we are called upon to bleed, either from the arm or by the application of leeches or cups over the affected part, and to repeat the operation as often as the symptoms continue urgent. The cinchonic preparations, in full doses, will then complete the cure. In all protracted cases of the disease, bleeding is of absolute necessity, if we desire to remove the sustaining causes, viz. either chronic inflammation of the liver or spleen, or of the gastro-intestinal mucous membrane, or sometimes all conjoined. The supervening of anasarca, abdominal dropsy, chronic bronchitis, or pleurisy, will demand similar treatment; and, until the local malady be removed, and the stomach clear of irritation, (of which we shall find the evidence in a tongue moist and divested of preternatural redness,) we cannot hope for any good from the bark and its kindred preparations. On the contrary, painful and protracted disease, variously masked, will too often be the consequence of their exhibition, where these rules are disregarded.

It is, perhaps, due to those who are sceptical of the value which ought to be attached to inferences from the phenomena of a disease, and its analogies to others, to mention that the principles and practice, which I have deemed it my duty to lay down in the treatment of intermittent fever, have been tried by the test of a tolerably long period of successful experience. Independent of the opportunities for observation of anterior date, my situation for the last eight years as a physician to the Philadelphia Dispensary, has rendered it necessary for me to treat hundreds of subjects of this fever, from all regions and of all ages, to say nothing of the no small number of cases in private practice, scarcely less varied in their symptoms and complications.—*North American Med. and Surg. Journal.)*

